# Supplementary material for: Determination of Nucleotide–Nucleotide and Nucleotide–Amino Acid Binding Interactions from All-Atom Potential-of-Mean-Force Calculations
Source: ACS Phys Chem Au. 2026 Jan 23;6(2):308–23. doi: 10.1021/acsphyschemau.5c00120 (PMC13022791; doi:10.1021/acsphyschemau.5c00120)
Supplement: Supplementary file 1 [file pg5c00120_si_001.pdf]

Determination of nucleotide-nucleotide and nucleotide-amino acid binding interactions from all-atom potential-of-mean-force calculations

Alejandro Feito[‡], Eduardo Pedraza[‡], Estefania Cuesta, Alejandro Castro, and Antonio Rey

Department of Physical Chemistry, Universidad Complutense de Madrid, Av. Complutense s/n, Madrid 28040, Spain.

Ignacio Sanchez-Burgos

Yusuf Hamied Department of Chemistry, University of Cambridge, Lensfield Road, Cambridge CB2 1EW, UK.

Rosana Collepardo-Guevara

Yusuf Hamied Department of Chemistry, University of Cambridge, Lensfield Road, Cambridge CB2 1EW, UK. Department of Genetics, University of Cambridge, Downing Street, Cambridge CB2 3EH, UK. and PhAsIca Biosciences S.L, Calle Velázquez, 27, 28001 Madrid, Spain

Andrés R. Tejedor*

Yusuf Hamied Department of Chemistry, University of Cambridge, Lensfield Road, Cambridge CB2 1EW, UK. and Department of Physical Chemistry, Universidad Complutense de Madrid, Av. Complutense s/n, Madrid 28040, Spain.

Jorge R. Espinosa[†]

Department of Physical Chemistry, Universidad Complutense de Madrid, Av. Complutense s/n, Madrid 28040, Spain. Yusuf Hamied Department of Chemistry, University of Cambridge, Lensfield Road, Cambridge CB2 1EW, UK. and PhAsIca Biosciences S.L, Calle Velázquez, 27, 28001 Madrid, Spain

‡These authors contributed equally

INTEGRATION OF THE PMF CURVES

To quantify the strength of the attractive interactions between nucleotides and/or amino acids beyond just the depth of the minimum, we computed the integral of the negative part of the PMF curves. This approach captures not only the minimum energy but also the width of the attractive region and any secondary minima.

Mathematically, the integral of the attractive region of the PMF, $U(r)$, is defined as:

$$I = \int_{r_i}^{r_f} U(r)\,\mathrm{d}r \quad \text{with } U(r) < 0 \tag{S1}$$

where $U(r)$ is the PMF as a function of the distance r between interacting sites and r_i and r_f are the bounds of the region where $U(r) < 0$, i.e., the attractive part of the curve.

In practice, given the discrete set of points (r_k, U_k) obtained from simulations, the integral was approximated using the trapezoidal rule:

$$I \approx \sum_{k=1}^{N-1} \frac{U_k + U_{k+1}}{2}\,(r_{k+1} - r_k), \quad \text{for } U_k, U_{k+1} < 0 \tag{S2}$$

where N is the number of discrete points in the attractive region.

The standard deviation of the integral, σ_I, was estimated by propagating the pointwise uncertainties σ_k associated with each PMF value:

$$\sigma_I = \sqrt{\sum_{k=1}^{N-1} \left[\frac{\sqrt{\sigma_k^2 + \sigma_{k+1}^2}}{2}\,(r_{k+1} - r_k) \right]^2} \tag{S3}$$

The resulting integral I thus provides a measure of the overall attractive interaction in the PMF well:

$$I \pm \sigma_I \quad (\text{kcal·nm/mol}) \tag{S4}$$

* at949@cam.ac.uk

† jorgerene@ucm.es

SUPPLEMENTAL FIGURES

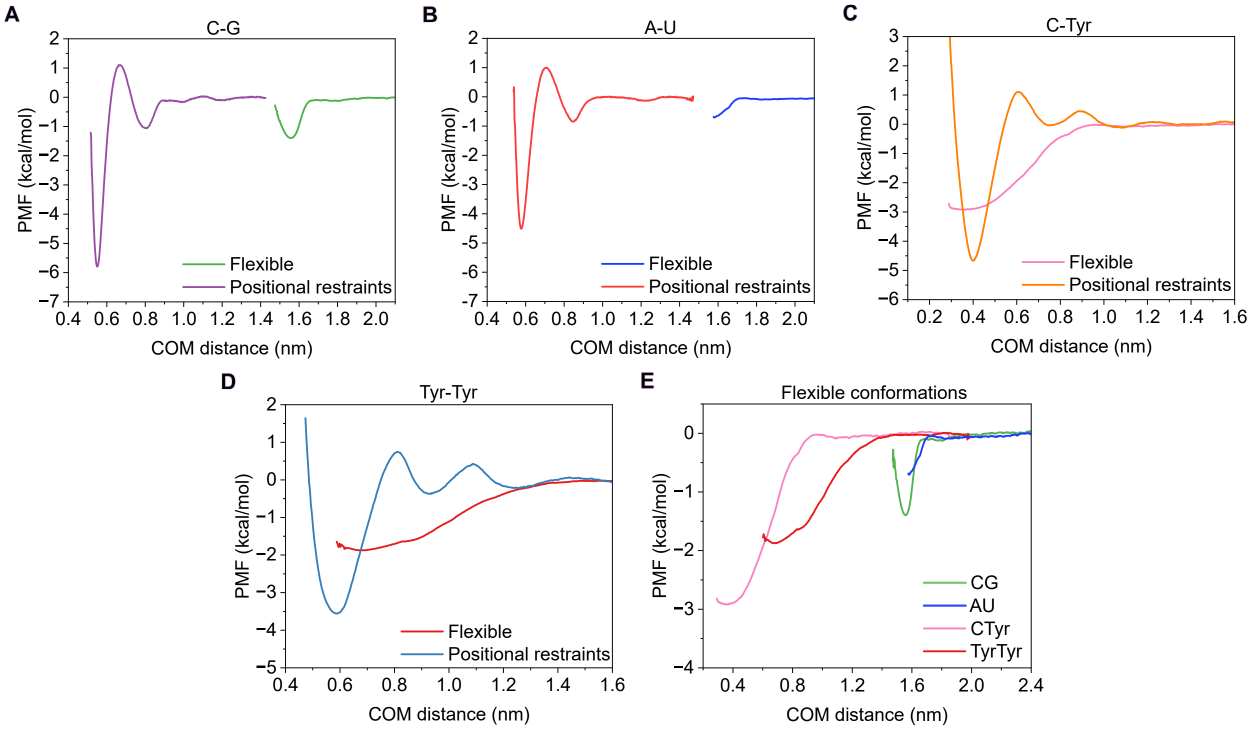

FIG. S1: Difference of atomistic potential of mean force (PMF) dissociation curve along the COM distance between the bases or side chain of the corresponding nucleotides or amino acids, respectively, at 300 K, 1 bar and physiological NaCl concentration (150 mM) in explicit solvent an ions with positional restraints in heavy atoms or only in the part of the molecule that is not confronted for the cytosine-guanine pair **(A)**, adenine-uracil pair **(B)** and cytosine-tyrosine pair **(C)** with the AMBER03ws force field and tyrosine-tyrosine pair **(D)** with the a99sb-*disp* force field. **(E)** Curve of PMFs for the flexible pairings. The center of mass (COM) and the positional restraints of these flexible conformations have been defined in the part of the molecule that is not confronted.

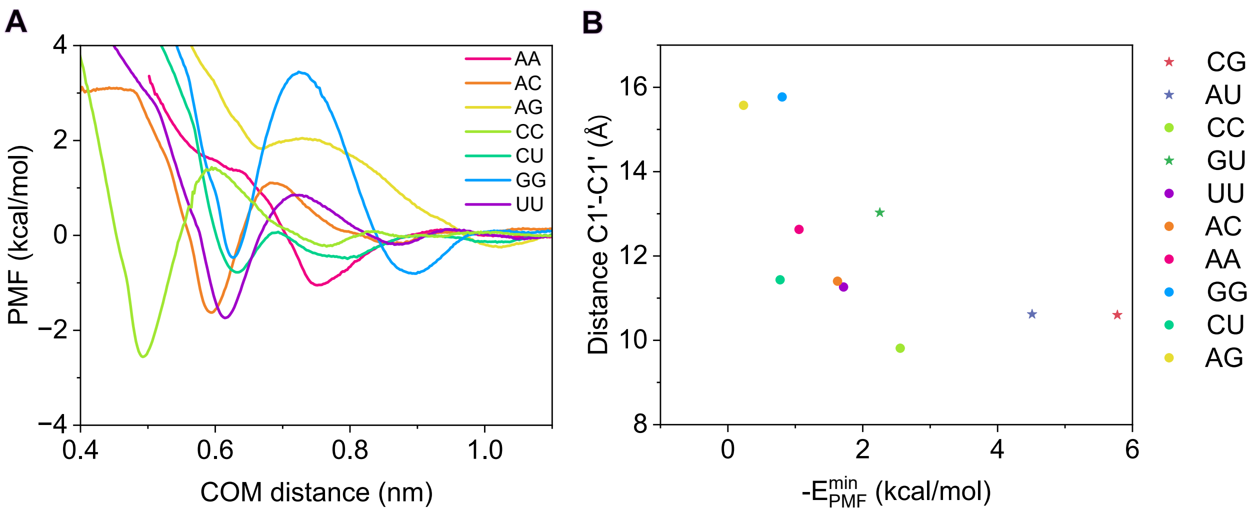

FIG. S2: (**A**) Atomistic potential of mean force (PMF) dissociation curve of the different nucleotides non-canonical pairs studied as a function of the center-of-mass (COM) distance between the bases of the corresponding pair using AMBER03ws force field. PMF simulations have been conducted at 300 K, 1 bar and physiological NaCl concentration (150 mM) in explicit solvent and ions. Nucleotides have been confronted with their bases. (**B**) Distance C1'-C1' vs. minimum energy ($-E^{\mathrm{PMF}}_{\mathrm{min}}$) of the PMF curve for the different pairs of nucleotides.

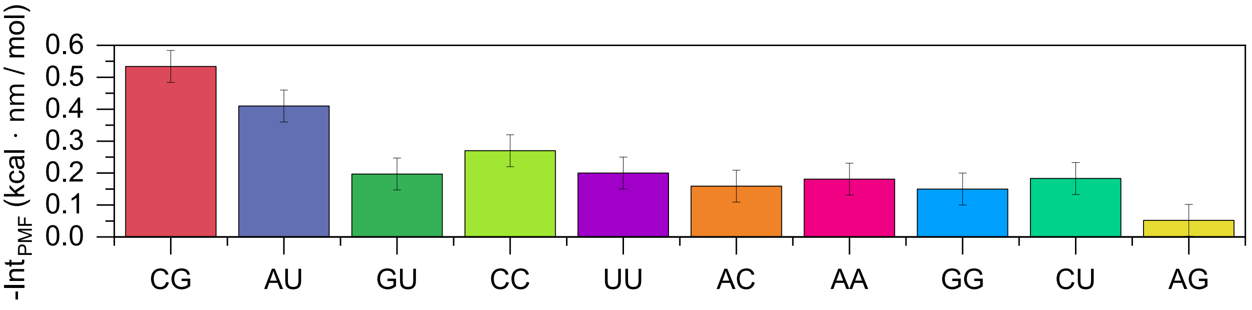

FIG. S3: Integrals of the attractive part of the corresponding PMF curves for canonical and non-canonical nucleotide pairs. All calculations were performed at 300 K, 1 bar and 150 mM NaCl with the AMBER03ws force field.

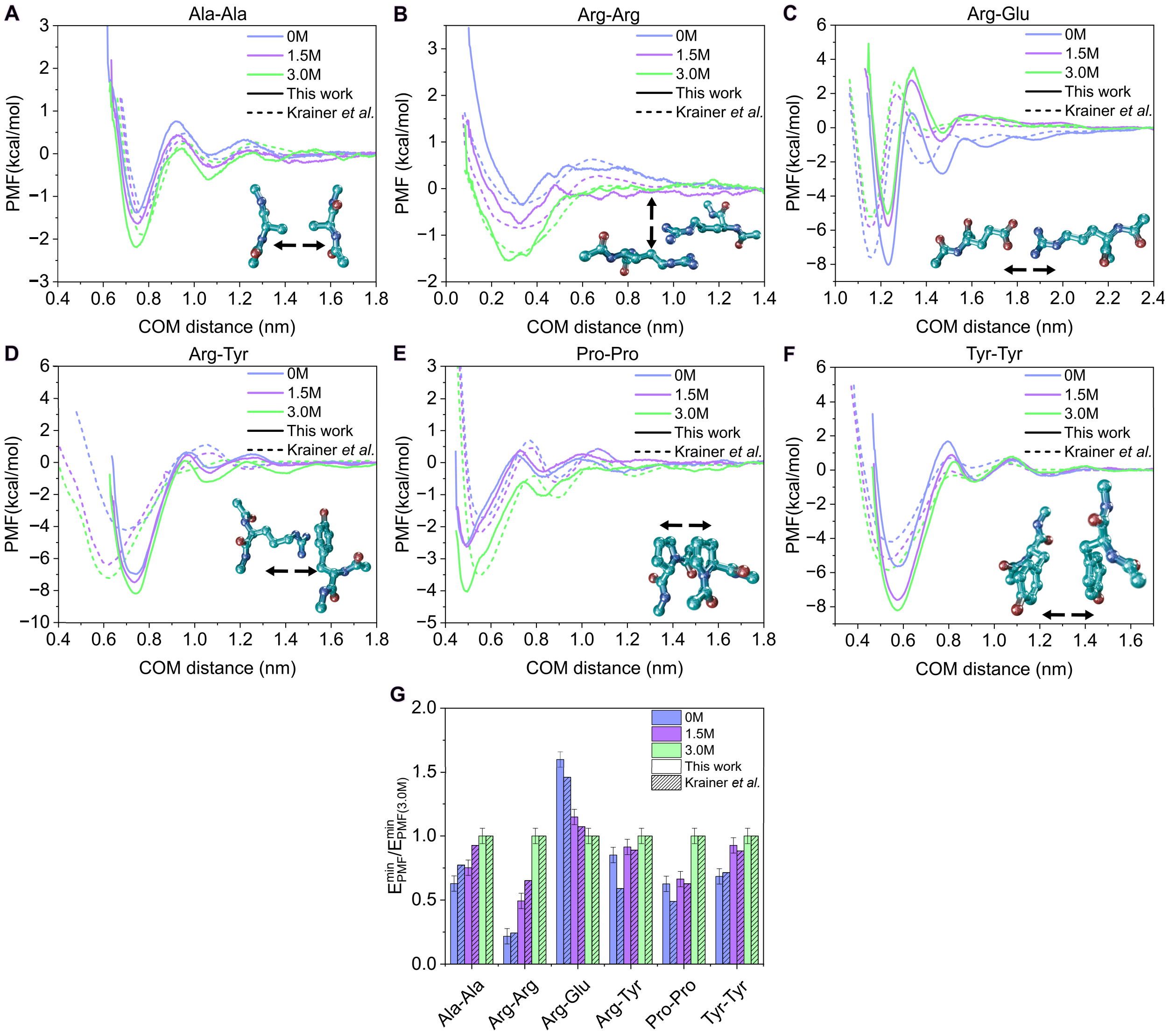

FIG. S4: Effect of salt concentration of NaCl 0 M (indigo), 1.5 M (violet) and 3 M (light green) on the atomistic potential of mean force (PMF) along the COM distance between the side chain of the corresponding amino acid pairs in explicit solvent and NaCl ions at 300 K and 1 bar as a function of the center-of-mass (COM) distance using AMBER03ws force field. The selected amino acids are alanine-alanine (**A**), arginine-arginine (**B**), arginine-glutamic (**C**), arginine-tyrosine (**D**), proline-proline (**E**) and tyrosine-tyrosine (**F**). The data from this work have been plotted in Figures **A-F** with a solid line and the results of Ref. [1] in a dotted line. In Figs **A-F** as an inset we have represented the different configurations of these pairs including arrows to indicate the dissociation direction. The minimum energy of the PMF normalized against the value of that pair at 3.0 M has been plotted for the different pairs in **G**. In this Figure the results of this work have been represented with solid bars, and the results of Ref. [1] with dashed bars.

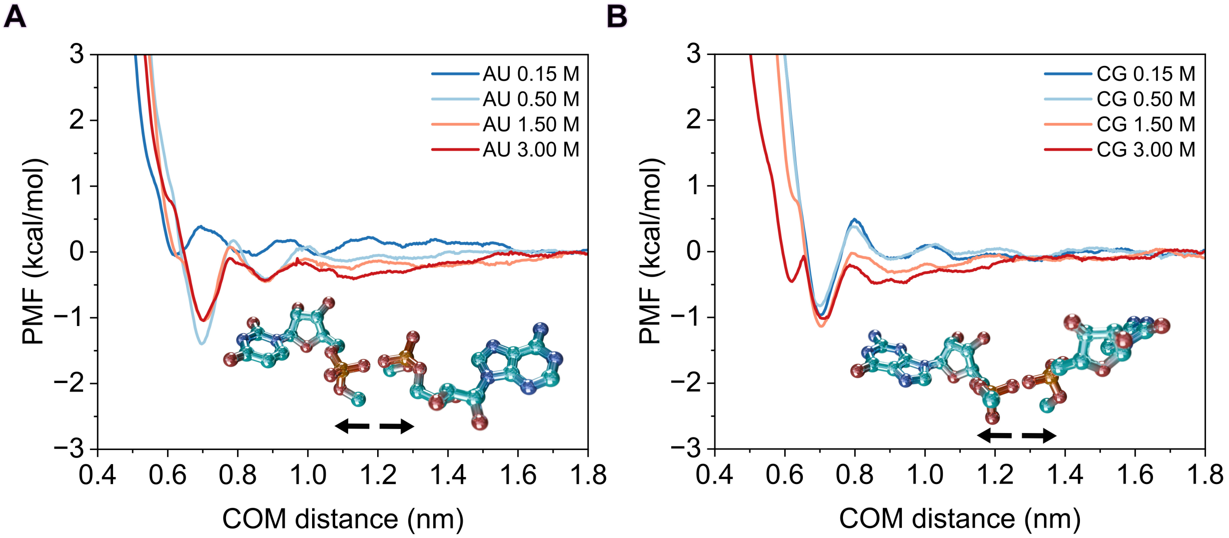

FIG. S5: PMF along the COM distance between the phosphate group of the corresponding nucleotides at different concentrations with AMBER03ws for the canonical nucleotide pairs AU (**A**) and CG (**B**), oriented via their phosphate groups. We include a representative snapshot of the different configurations for the pairs, including arrows to indicate the dissociation direction.

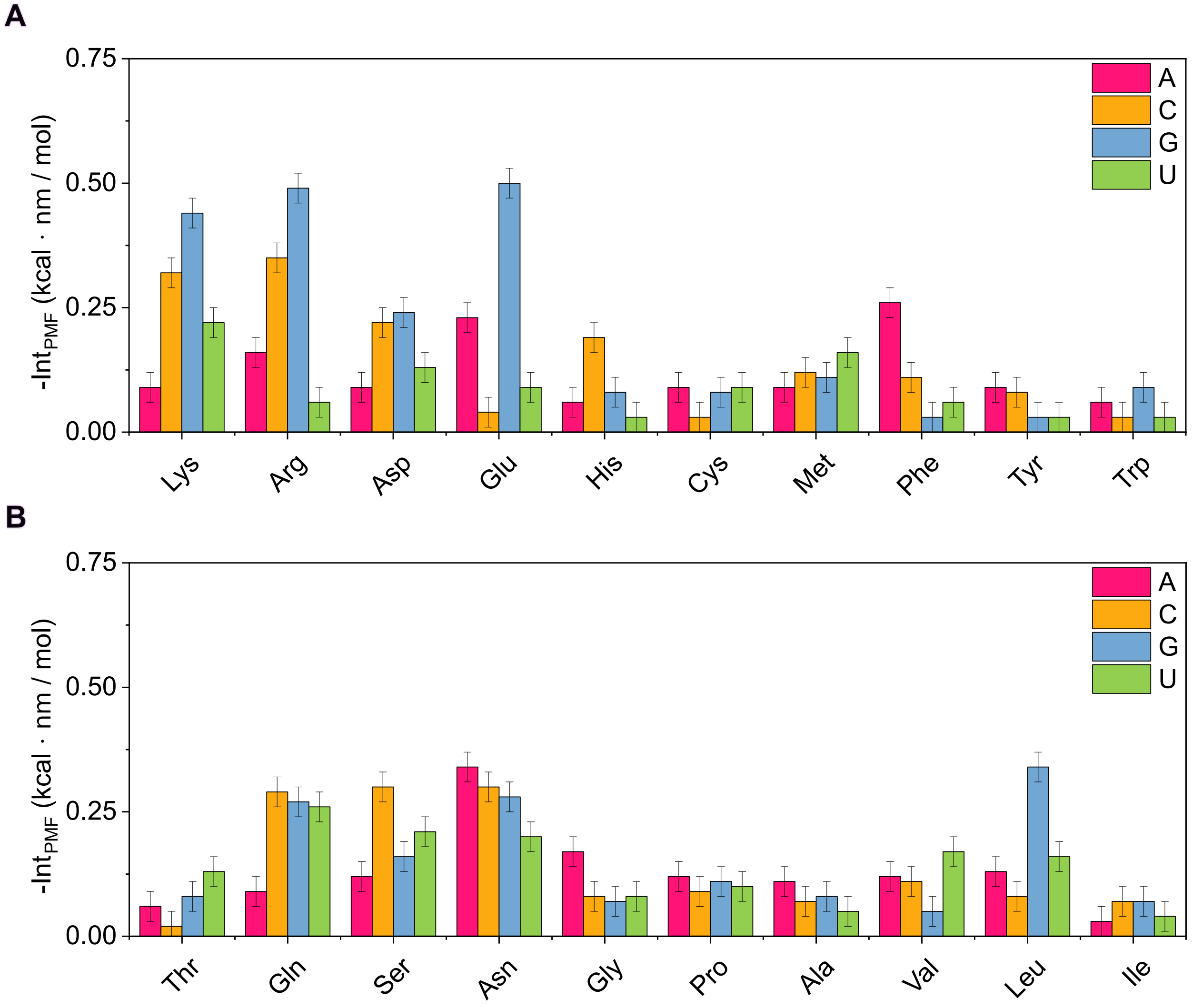

FIG. S6: Integrals of the attractive part of the corresponding PMF curves of each nucleotide–amino acid pair are shown. Amino acids (**A**) Lysine, arginine, aspartic acid, glutamic acid, histidine, cysteine, methionine, phenylalanine, tyrosine, and tryptophan, (**B**) threonine, glutamine, serine, asparagine, glycine, proline, alanine, valine, leucine, and isoleucine with the nucleotides adenine (pink bars), cytosine (yellow bars), guanine (blue bars), and uracil (green bars).

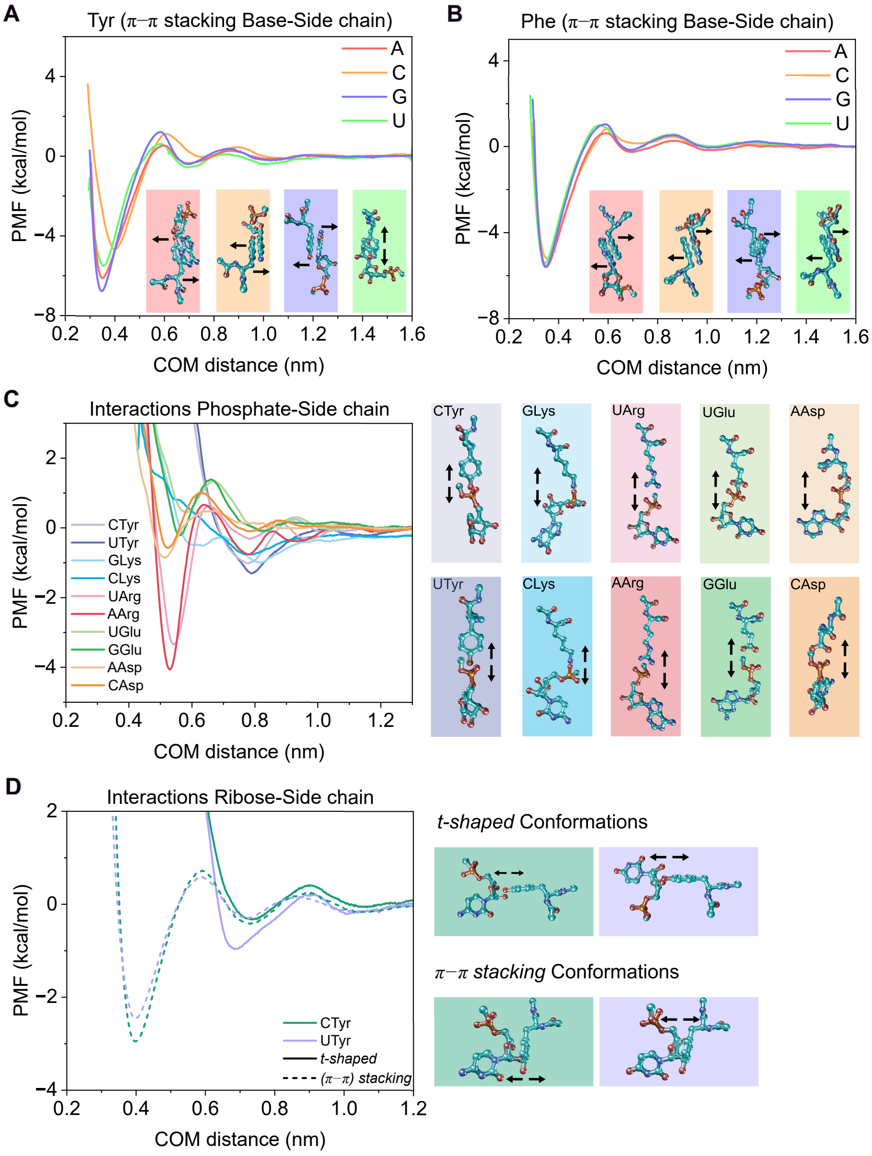

FIG. S7: PMF dissociation curve at 300K and 150 mM of NaCl in explicit solvent an ions using AMBER03ws force field between selected nucleotides and amino acids. Interaction via π-π stacking of the nucleotide's base and the amino acid's side chain for the different nucleotides with tyrosine (**A**) and phenilalanine (**B**). (**C**) PMF curve of the confronted nucleotide's phosphate and the amino acid's side chain between different nucleotides and amino acids. (**D**) PMF curve of the confronted nucleotide's ribose and the amino acid's side chain between the tyrosine with cytosine and uracil. In solid curves we plotted the t-shaped conformations and in dotted curve the π-π stacking conformations. On the right side of the panels (**C**) and (**D**) we include representative configurations of the pairs including arrows to indicate the dissociation coordinate.

REFERENCES

---

[1] G. Krainer, T. J. Welsh, J. A. Joseph, J. R. Espinosa, S. Wittmann, E. de Csilléry, A. Sridhar, Z. Toprakcioglu, G. Gudiškytė, M. A. Czekalska, W. E. Arter, J. Guillén-Boixet, T. M. Franzmann, S. Qamar, P. S. George-Hyslop, A. A. Hyman, R. Collepardo-Guevara, S. Alberti, and T. P. J. Knowles, Reentrant liquid condensate phase of proteins is stabilized by hydrophobic and non-ionic interactions, Nature Communications **12**, 1085 (2021).
